# Notes and Sketches, Preparatory to a Treatise on Diseases of the Tropics

**Published:** 1860-03

**Authors:** G. Van Arcken

**Affiliations:** Bogotá, New Grenada


*Notes and Sketches preparatory to a Treatise on Diseases of the Tropics.* By G. Van Arcken, M.D., Bogota, New Grenada.

*Prostatitis.*—Chronic inflammation of the prostate gland, as a consequence of neglected or ill-treated gonorrhœa, is a very common disease in the tropics. As it mostly happens in old and debilitated subjects, much benefit may be derived from tincture of bark, with small doses of bi-chloride of mercury or of iodide of potassium.

If the disease partakes more of an acute character, the warm hip-bath, fomentations, poultices and leeches, do great good.

In some extreme cases, where the urine is passed with great difficulty, and dilatation by bougies has been found impracticable, cauterization, by means of a small quantity of nitrate of silver, either in powder or in ointment, may be resorted to, and repeated according to circumstances.

A liberal diet should be allowed in all these cases, and an occasional tonic infusion or tincture given.

*Syphilis.*—In all the history of mankind, there never was a more shameful untruth told, a more grievous wrong committed, than when some medical humbug raised the story of the American origin of the venereal disease, which, they say, was first imported from the New World by the crews of Columbus. I have often wondered how men of high standing in the medical world could possibly countenance such a gross absurdity.

Wherever prostitution is restricted to a few women among a crowd of men, there various classes of foul diseases must be generated

The vaginal secretions of many a healthy woman will sometimes produce either a gonorrhœa, balanitis, or even slight ulcers, especially in those persons the mucous membrane of whose genitals is so delicate as to be affected by the slightest resistance which the vagina may offer to the entrance of the male organ. But these cases are easily distinguished by their benignity; a strict attention to cleanliness, rest, and a slightly astringent lotion, being sufficient to effect a cure.

In hot countries, where people are always more or less lasciviously inclined, a slight urethral discharge may be brought on by venereal excesses, which may afterwards be converted into gonorrhœa by intercourse with a woman, who, although chaste, suffers from some slight vaginal discharge.

At any rate, I consider it my duty to take a decided stand against the pretended American origin of venereal diseases, simply because, in all my travels in North, Central, and South America, I have always found the pure Indian breed a remarkably healthy race, entirely free from syphilis; while, on the contrary, the mixed races are a continual prey to it.

As I have already remarked, the treatment of primary syphilis is much more easy in hot than in cold countries. Within the first fourteen days, an occasional dose of calomel and jalap, followed by epsom salts, and emollient applications to the ulcer, is all that is required. If the disease is of longer standing, and the edges of the ulcer already hard and callous, the mildest mercurial preparation, hydrargyr. c. creta, may be given in two-grain doses, every night, for ten or twelve days. There is hardly ever any difficulty in curing the ulcers, and, as a general thing, the soothing applications bring about the desired effect much more promptly than caustics.

With regard to secondary and tertiary syphilis, although it is true that in some constitutions the corrosive sublimate, in tincture of bark, will act like a charm, still my general experience has gone against mercurials, and I now consider them, in this peculiar class of diseases, to say the least of them, very fallacious remedies.

Most of these cases can be completely cured by a purgative regimen, continued with intervals for four or six weeks; and if afterwards any lingering disposition remains behind, the best remedy I know of is Fowler's Solution, in ten-drop doses, twice daily for a few weeks.

Formerly I had great faith in the vegetable remedies, so extolled against this disease by some practitioners, especially sarsaparilla, guiacum, quassia amara, and sassafras; but after having given them a fair trial in several cases, where mercury and iodine were not admissible, I found them to be nothing but trash, on which money and time were thrown away.

The arsenic has failed only twice in my hands, and in those two cases I effected a cure by tincture of tartrate of iron and potash, in twenty-drop doses, three times daily for about six weeks, the drops being taken in a spoonful of old Hock.

*Cystitis.*—The acute and the chronic form of this disease form, with regard to frequency, the most complete contrast. The acute, very rare indeed, has but twice come to my notice; the treatment does not differ materially from that pursued in colder latitudes.

The chronic form is of so frequent occurrence, that hardly a day passes without my being consulted about some form or other of this most fatal disease. I say most fatal disease, because the patients have seldom sufficient endurance to remain in the charge of a physician until a complete cure is effected; and being only partially relieved, the first cold or wetting of the feet is sufficient to bring on a new and aggravated attack. This happens over and over again, and at length the patient, worn out by want of sleep, agony and hectic, dies a most wretched death.

I take it for granted that all my readers know how to mark the exact diagnosis of a chronic or latent inflammation of the mucous membrane of the bladder.

The first attack of this disease is but rarely characterized by grave symptoms, and frequently it commences so insidiously that only by some accident the patient discovers a cloudy state of the urine, which alarms him.

For this I prescribe, first an infusion of senna, with salts, and afterwards, every night and morning, a Seidlitz powder. This, together with an occasional tepid bath, and a vegetable diet, is sufficient to effect a cure in from 15 to 20 days.

Let us suppose now that the patient, seeing himself relieved at the end of eight days, thinks, as so many do under similar circumstances, himself cured, and goes to work again.

A few months afterwards he gets by chance a severe wetting, and on the next day he feels a strange sensation on voiding his urine; that is to say, the moment he commences to pass it, a strange sensation creeps over him, which gradually increases, until it amounts to a positive deep-seated pain in the bladder. The same medicines and diet are again taken hold of, and at the end of four weeks the patient goes to work again, being directed to take, for at least six weeks, three times daily, ten drops of the muriated tincture of iron.

But as this medicine is not very agreeable to take, after using it two or three days, in a fit of rage, he pitches it out of the window.

Still, he never feels himself entirely well, and on certain days an aversion to walk or move comes over him, which gradually increases, until, according to his own saying, he is too lazy to get out of bed.

Matters now grow rapidly worse; in the urine there appears a thick, muco-purulent deposit, and occasionally the whole is tinged with blood.

Extract of uva ursi, buchu, catechu, and opium are now prescribed, and after a partial re-establishment has been effected, the muriated tincture of iron is taken up again.

Perhaps the patient goes on improving for some time, but sooner or later a new symptom appears, which rather annoys him, to wit: he cannot retain his urine longer than one hour at furthest. The pain after passing urine becomes worse; and now, when the first drops of it pass the urethra, it scalds like fire.

Venetian turpentine, balsam of Peru, and other remedies of a similar nature, are now employed; at first they produce a temporary relief, and then the disease continues its course. At length the attending physician sees the uselessness of all internal remedies, and proposes injections into the bladder. If the patient be a male—good; but if a female, their universal answer is, Rather die! And die they do, soon after, of exhaustion, want of sleep, and hectic.

The remedies for injections are those used for diseases of the urethra.

I generally commence with barley-water, then diluted liq. plumb. acetat., and go then over to sulphate of zinc and nitrate of silver. In those cases where injections are resorted to at the right time, a permanent cure is sometimes effected; but those cases are the exception; the rule is death in from two to four years' time from the first attack of the disease.

*On Fevers in general.*—It has been truly said that America is the home of fevers of every possible description; and if both the polar extremities are comparatively free from them, so much more do they abound in the tropics.

No disease whatever, the slightest ailment, a toothache, for instance, let it continue for a week, and soon the experienced physician will see the pain become periodical, and assume in a few days more the type of an irregular intermittent fever.

It is this which mostly astonishes newly-arrived physicians, for their general opinion is, that all practitioners become here empirics; while, on the contrary, it is experience which teaches us to employ quinine in a great many cases "*for which it is not recommended in the books.*" Quinine is the great remedy of America; but it is a remedy that few know how to give with advantage. As my space is limited, I must abstain from giving my opinions about its uses and manner of employing it, except in cases of genuine fevers, where I shall state my manner of administering it in a few words.

*Intermittent Fever.*—As in all other countries, this is the kind of fever which most frequently occurs. It is remarkable only for its obstinacy; in the majority of cases, quinine in scruple doses will break it up; the same remedy must afterwards be continued, partly to prevent a relapse, partly to assist nature in performing the necessary amount of repair. Three grains every night and morning are sufficient for this purpose; but it must be continued at least three weeks.

In cases of several months' standing, although quinine will break the fever up, alone it is seldom able to cure it. In those cases, I prefer the new preparation, called Chinioidine, of which I give, twice daily, three grains, with an equal quantity of quinine. I continue this for about six weeks, suspending its use once every fourteen days, for the purpose of giving a strong dose of epsom salts. After this, I let the patient take, for some three or four weeks, daily, six grains of ammoniate of iron, to correct any obstruction in the portal system; and should the spleen be enlarged, employ iodide of lead externally.

It is but seldom that the plan proposed above has failed me, and in those few cases, Fowler's Solution, in ten-drop doses, continued for several months, effected a cure; still, I do not like to employ it, for it mostly deranges digestion to a considerable extent, a very disagreeable attendant in cases of fever.

*Congestive Fever.*—Formerly, I thought there was nothing like the congestive fever of New Orleans and the surrounding countries, but my travels on the plains of Venezuela have convinced me that the latter country decidedly deserves the not very enviable reputation of being the home of fevers; congestive fever foremost.

In the States, very few cases die in the second chill, most of them in the third. In the plains of Venezuela most all die in the first chill, and but few survive to sink under the first blow of the second.

It may be asked, What can a physician do against so terrible a disease? That is the same question which I asked myself and my confrères, when first arriving in that woe-begotten country. My confrères answered me with a shrug of the shoulders.

Still, I did not despair, and if I could not save all my patients, at least I have saved the majority of them.

This is my plan of treatment:

The disease being of so fatal a nature, my chief aim is to economize time, and to bring about an ample reaction without delay. For this purpose, I take a spoonful of mustard, common salt, or some ipecac, put it in a tumbler of tepid water, and make the patient take it. Ten minutes after that, I commence slowly to roll the patient from side to side, and if this does not cause him to vomit pretty soon, then—*ultima ratio*—I push two fingers down his throat.

This is followed immediately by copious vomiting, which must be encouraged by plenty of tepid water, with some salt in it.

The effect of a vomit at such a critical moment is to make the fever jump from the first state to the third, entirely superseding the second. Directly after the vomiting has ceased, the patient commences to transpire most copiously; and, as soon as this shows the slightest tendency to stop, then is the time to give quinine.

The vomitive mostly leaves the stomach in rather a tender state, which makes it impossible to give any medicine internally; for that reason I dissolve, say half an ounce of pure quinine in four ounces of sulphuric ether, and to facilitate the dissolving, add half an ounce of liquor ammoniæ. One-half of this solution is rubbed in on the abdomen and under the arm-pits of the patient; the same is repeated with the remaining half about six hours afterwards.

This is all the treatment that is required. In some few cases there comes on, the second or third day afterwards, a trifling chill; but if, on the day after the first attack, there be no quininism, a scruple of quinine should be taken in some strong coffee, and the dose repeated six hours afterwards; and if due attention is paid to this, there is no danger whatever of relapse. Let nobody be scared at the tremendous dose employed externally; I lost several patients before I became bold enough to use it, thinking that one or two drachms were sufficient.

*Yellow Fever.*—I refer my readers to the May number of the MONTHLY, for 1858, in which they will find a description of my experience in this disease during my stay at Port-au-Prince; I have nothing to add to the treatment which I have minutely described in the article alluded to.

*Typhoid Fever.*—About this disease, so frequent in the tropics, I have nothing new or important to say, except to detail my method of giving quinine.

It is a fact known to every practitioner, that in no disease is there such a diversity of opinion with regard to the employment of quinine, as in this. I am not vain enough to flatter myself with the idea of having discovered the cause of this diversity of opinion, based, of course, upon different results; but I can state, that since I have adopted my present plan, not one-fourth of my patients suffering from this disease have been over one week in bed. My plan is as follows:

In all fevers of a typhoid character, the functions of the alimentary canal, and of the skin, are, from the beginning, entirely prostrated. This is the reason why no remedy, especially quinine, whether given internally or externally, can produce its effect, for not a particle of it enters the circulation.

My plan of treatment, then, is to rouse both to increased action.

On the first day, I give the following:

℞.—Sulphatis magnesiæ, . . . . . ℥ij.<br>

Tartari emetici, . . . . . grs. viij.<br>

Aquæ fontan., . . . . . . ℥viij.

M. et solve.

S.—Give two table-spoonfuls every fifteen minutes, until copious vomiting has taken place; after that, repeat the same dose only every two hours.

The second and third day I give:

℞.—Decoct. seminis hordei, . . . . . ℥vij.<br>

Vini tartari emetici, . . . . . ℥j.<br>

Liq. ammon. anisat., . . . . ʒij.<br>

Syrup. cort. aurant., . . . . ℥j.

M.

S.—A wine-glassful every hour.

Under the use of these remedies, the stomach and bowels are effectually cleaned out, and the edges of the tongue, formerly covered with a thick, white fur, get rather red, and the skin, from being hot and dry, has become soft and moist.

The next morning, that is, on the fourth day, is my time of election for giving quinine, the patient being now what I call properly prepared for its action.

I give a scruple of it internally, in a little strong coffee, which makes it sit better on the stomach. At the same time, I dissolve one drachm in one ounce of sulphuric ether, adding one fluid drachm of liquor ammoniæ. This must be rubbed in on the abdomen and under the arm-pits.

If, on the next morning, there be quininism, good; if not, I give another scruple internally.

After this, I let my patient have plenty of good broth, and three days afterwards he is up and about.

*Bilious Fever.*—The treatment against this disease has nothing to distinguish it from the way my confrères on the shores of the Mississippi handle this enemy of humanity.

I make it a rule to give an alterative mercurial pill every three or four days, for at least six weeks after an attack of bilious fever; for experience has shown me that it is almost always accompanied with more or less inflammatory action in the liver, and a subsequent qualitative and quantitative abnormity in the functions of the hepatic secretion.

In cases where no attention is paid to this precaution, the fever is sure to return, or bring about a chronic hepatitis.

*Acclimatization Fever.*—The letter which I directed a short time ago to the editors of this Journal, and is published in the May number of the MONTHLY for 1859, explains my views on this particular kind of fever.

*Hepatitis.*—This is undoubtedly the disease which most frequently occurs in the tropics. The elevated temperature acts as a direct stimulus to the liver, and predisposes it to inflammatory action.

Acute hepatitis is rarely a fatal disease. Chronic hepatitis, on the contrary, is but seldom radically cured; the most prominent and troublesome symptoms may disappear for a time; still, sooner or later, they return with renewed strength, and at last the patient sinks, unless, indeed, he be carried off by an intercurrent disease; a thing which often enough happens.

The reason why hepatitis is so rarely radically cured is the horror in which the natives keep anything related with medicine. As soon as the most grave symptoms have disappeared, they fancy themselves cured, and refuse to take any more medicine.

A second, and by no means unimportant cause, is the entirely out-of-the-way treatment pursued by natives, and by most of the foreign practitioners in this country.

The natives, with their limited medical education, are almost all of them followers of the new French school; they let the time pass with insignificant tisanes and other medicines, which cannot hurt a baby, but make no impression whatever on the disease. Mercury, even when given in small alterative doses, is a horror to them.

The foreign physicians, on the contrary, being most of them "*irregular graduates of nature's school of experience,*" fall into the opposite extreme, of giving mercury in abusive doses. This is especially the case with Englishmen, who know of nothing else than their eternal blue pill.

Now, if it is difficult to cure a case of hepatitis without mercury, when this remedy is abused, a cure is entirely out of question; and in many a case do I believe that the curative powers of nature have been paralyzed, and the disease gone on to a fatal termination, just because of the brainless administration of mercury.

In cases of acute hepatitis, after the antiphlogistic regimen has put aside the most dangerous symptoms, calomel in one-grain doses, repeated every two or three days, should be resorted to. Of course it must be suspended as soon as the gums commence to get sore, for profuse salivation is decidedly injurious.

The intermediate time may be usefully employed with giving laxatives, acid mixtures, and making use of repeated blisters.

This, together with a suitable diet and an occasional tepid bath, is sufficient to effect a cure in nine cases out of ten.

Chronic hepatitis needs pretty much the same treatment, only mercury should be given more sparingly, and frequent recourse had to drastic purges.

A remedy, the use of which has of late fallen into undeserved discredit, is the muriate of ammonia. It well deserves a fair trial in all cases where mercury is inadmissible, because suppuration has already taken place. It appears to act as an alterative, gently stimulating the excretory power of the liver; at any rate, under its protracted use, and alternate drastic purges, I have frequently observed most difficult cases of chronic hepatitis to improve so much, that no physical sign could enable me to detect any tenderness or increase of size in the liver.

Externally, the hip-bath, with nitro-muriatic acid, and the long-continued use of a plaster composed of gum-ammoniac and mercury, will mostly be productive of much good. Counter-irritation, by blistering, tartarized antimony, and setons, has been much lauded by some practitioners, although I think that the good they do is more than counterbalanced by the annoyance they create. If the patient wants to try them, good; but I never propose them of myself.

*Carate.*—This name is given to a new and quite peculiar disease of the skin, which is endemic on the shores of the rivers Magdalena, Cauco, Atrato, Qulio, Catatumbo, Apure, Meta, and Orinoco; all of them traversing the northern part of the South American Continent.

I refer my readers to the April number of the MONTHLY for 1858, in which they will find a detailed description of this disease.

*Pemphigus.*—Pemphigus, or Pompholyx benignus, as Willan calls it, is in the tropics by no means so trivial a disease as in colder latitudes. The classification of it into chronic and acute is quite useless here; for all cases of it commence with acute symptoms, which gradually take the chronic form. In fact, I am almost inclined to think that this disease is only curable after it has passed from the acute to the chronic form.

If called to a case of acute pemphigus, I limit myself to giving some strong purges, and after that try to bring about a gentle diaphoresis. If the eruption is rather tardy in making its appearance, I order one or two baths with sulphuret of potass, or some frictions with any irritating substance. As soon as the eruption is fairly out, every trace of fever leaves, and the chronic state of pemphigus commences.

The specific against pemphigus, and almost any disease of the skin, is corrosive sublimate; and if this remedy has not been equally successful in all hands, that may in part depend upon the manner in which it is administered.

In this country, and the tropics in general, it is almost impossible to find a family entirely exempt from syphilitic taint. Having such a foundation, it is not at all strange that pemphigus should frequently be accompanied by tertiary symptoms; all of which, together with the original disease, soon give way under the use of sublimate. My favorite way of using it is the following: I order a solution to be made, containing about five grains of it to every ounce of water. With this all the pustules that are not yet ripe are to be rubbed, until the skin becomes quite red: this causes them to abort. Those that are already filled with water or pus should have their cuticle removed by one sweep with a pair of sharp scissors; the liquid will then escape, and the bottom of the bullæ should be wetted with the solution.

This entirely external plan of treatment will still sometimes produce salivation; of course, the use of the solution should be immediately suspended, and not taken up again until the mouth is well. It seldom requires longer than a fortnight to cure a most inveterate case of pemphigus after this manner of treatment.

*Spinal Irritation.*—Under this term I understand any morbidly-increased sensibility of the medulla spinalis, either entire or of a part of it, characterized by tenderness, upon pressure, of one or more of the spinous processes of the vertebræ.

The symptoms of this disease are immensely diversified; much more so in the tropics than in colder climates; and frequently appear to have so little connection with the medulla, as to be misunderstood for a long time, to the great injury of the patient.

Already several times have I been called to patients who have been for a long time under treatment for asthma, hepatitis, disease of the kidneys or bladder, when, after a thorough examination, I had no difficulty in tracing the disease to the medulla spinalis.

Diseases of this kind are so frequent, that I am in the habit of running my hand over the whole column of spinous processes, as soon as I find the least difficulty in making my diagnosis in some pretended disease of the thoracic or abdominal viscera, and most generally do I find a tender vertebra.

A pathognomonic symptom of great value, but which has never yet been brought forward in its true light, is that highly changeable state of the urine—being now perfectly clear and limpid, having to morrow a brick-dust deposit, on the day following a fatty coat on the top, and on the next day being quite clear again.

Whenever I find this state of the urine, although my first and second examinations detect no tender vertebra, I still commence my usual treatment for spinal irritation, and sooner or later, either by accident, or as a natural course of the disease, the tender spot is discovered, which need not be the spinous process, although in the majority of cases it is.

My treatment in this disease is very simple: drastic purges, when occasion requires it; mercury in small alterative doses, say one grain of calomel every three or four days; counter-irritation of all kinds; and opium, to allay pain.

After the most grave symptoms have disappeared under this treatment, then the exhibition of the tartrate of iron and potash will do the rest. Of all the iron preparations, this is my favorite, because it neither heats nor constipates, as all the others do, nor has it got the horrible inky taste of most of them.

This disease is sometimes complicated with gout or rheumatism, with which it may be easily confounded by practitioners of little experience.

Whenever such a complication exists, an occasional dose of iodide of potassium should be given in colchicum wine, and continued until the spinal disease has been brought back to its origin.

As a general rule, sulphurous baths, followed by frictions with some warm aromatic spirits, are highly beneficial in all cases of spinal irritation.

